# Chinese My Trauma Recovery, A Web-Based Intervention for Traumatized Persons in Two Parallel Samples: Randomized Controlled Trial

**DOI:** 10.2196/jmir.2690

**Published:** 2013-09-30

**Authors:** Zhiyun Wang, Jianping Wang, Andreas Maercker

**Affiliations:** ^1^Department of PsychologySchool of PhilosophyWuhan UniversityWuhanChina; ^2^Department of PsychologyUniversity of ZurichZurichSwitzerland; ^3^School of PsychologyBeijing Normal UniversityBeijingChina

**Keywords:** Web-based, stress disorders, traumatic, randomized controlled trial, self-help

## Abstract

**Background:**

Guided self-help interventions for PTSD (post-traumatic stress disorder) are a promising tool for the dissemination of contemporary psychological treatment.

**Objective:**

This study investigated the efficacy of the Chinese version of the My Trauma Recovery (CMTR) website.

**Methods:**

In an urban context, 90 survivors of different trauma types were recruited via Internet advertisements and allocated to a randomized controlled trial (RCT) with a waiting list control condition. In addition, in a rural context, 93 survivors mainly of the 2008 Sichuan earthquake were recruited in-person for a parallel RCT in which the website intervention was conducted in a counseling center and guided by volunteers. Assessment was completed online on a professional Chinese survey website. The primary outcome measure was the Post-traumatic Diagnostic Scale (PDS); secondary outcome measures were Symptom Checklist 90-Depression (SCL-D), Trauma Coping Self-Efficacy Scale (CSE), Post-traumatic Cognitive Changes (PCC), and Social Functioning Impairment (SFI) questionnaires adopted from the My Trauma Recovery website.

**Results:**

For the urban sample, findings indicated a significant group×time interaction in post-traumatic symptom severity (*F*_1,88_=7.65, *P*=.007). CMTR reduced post-traumatic symptoms significantly with high effect size after one month of treatment (*F*_1,45_=15.13, Cohen’s *d*=0.81, *P*<.001) and the reduction was sustained over a 3-month follow-up (*F*_1,45_=17.29, Cohen’s *d*=0.87, *P*<.001). In the rural sample, the group×time interaction was also significant in post-traumatic symptom severity (*F*_1,91_=5.35, *P*=.02). Post-traumatic symptoms decreased significantly after treatment (*F*_1,48_=43.97, Cohen’s *d*=1.34, *P*<.001) and during the follow-up period (*F*_1,48_=24.22, Cohen’s *d*=0.99, *P*<.001). Additional outcome measures (post-traumatic cognitive changes, depression) indicated a range of positive effects, in particular in the urban sample (group×time interactions: *F*_1,88_=5.32-8.37, all *P*s<.03), contributing to the positive evidence for self-help interventions. Differences in the effects in the two RCTs are exploratorily explained by sociodemographic, motivational, and setting feature differences between the two samples.

**Conclusions:**

These findings give support for the short-term efficacy of CMTR in the two Chinese populations and contribute to the literature that self-help Web-based programs can be used to provide mental health help for traumatized persons.

**Trial Registration:**

Australia New Zealand Clinical Trials Registry (ANZCTR): ACTRN12611000951954; https://www.anzctr.org.au/Trial/Registration/TrialReview.aspx?ACTRN=12611000951954 (Archived by WebCite at http://www.webcitation.org/6G7WyNODk).

## Introduction

Post-traumatic stress disorder (PTSD) is a common mental disorder after trauma. Although suffering from severe distress, many people with PTSD fail to ask for help from mental health professionals, especially in rural areas where people have more difficulties accessing traditional mental health services due to cost, time, geographic constraints, and stigmatization [1-2]. In recent years, the Internet has been adopted as a valuable tool to deliver mental health services to large populations [3-4]. Different Internet-based intervention programs have been developed to help people recover from PTSD [5]. Among them, some provide self-help Web-based interventions for users without support from therapists, like the programs examined by Hirai and Clum [6] and Benight, Ruzek, and Waldrep [7]. Other programs offer interventions for people with PTSD through the Internet with therapists involved to give instructions and feedback to the users, like Interapy [8-9]. These programs have been examined in American and European countries and have shown significant effects in reducing people’s traumatic stress-related distress [8,10]. However, few programs have been developed for and tested in Asian populations.

Recently, PTSD has gained much attention from public and mental health professionals in China. Based on the literature, a significant proportion of people suffered from traumatic distress after traumatic events, including earthquakes, floods, and traffic accidents [11-12]. However, few people got help from mental health professionals to deal with their trauma-related problems [13]. A major obstacle to people’s mental health help-seeking behavior is the lack of available professionals in China, especially in rural areas [14]. The number of qualified mental health professionals is small, even in large cities like Beijing and Shanghai [15]. Other main factors that hinder Chinese people’s mental health help seeking include fear of stigmatization, lack of information on mental illnesses and psychotherapy, confidentiality, etc [15-16]. The Internet thus offers a useful way to improve mental health services for people after trauma in China.

The current study aims to build a Chinese Web-based self-guided intervention program for traumatized persons and to test its effectiveness on Chinese populations. Two modalities of application of the intervention were involved: an unsupported use with preliminary urban clientele and modified use where clients were supported technically during the intervention by volunteers in a rural area. To test the effectiveness of the Chinese My Trauma Recovery (CMTR) program, the current study adopted a randomized controlled pre-, post-, and 3-month follow-up trial design (ACTRN12611000951954) in a two-arm design (urban/unsupported vs rural/supported). It was expected that participants from the two treatment groups would show significant improvement in PTSD symptoms and general mental health compared to the respective waiting list groups. Explorative post hoc analyses compared the effect sizes of the two arms of the study (urban/unsupported vs rural/supported).

## Methods

### Materials

**Figure 1:**
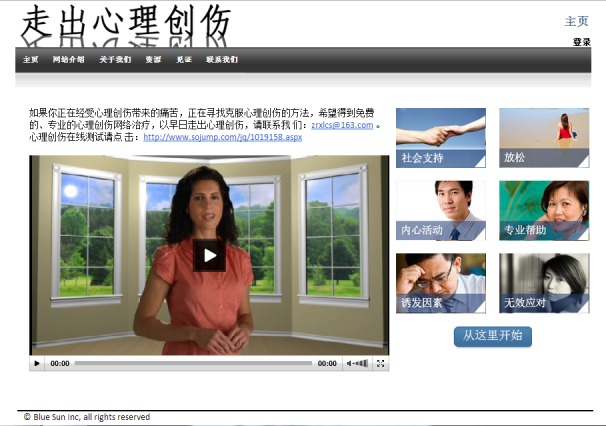
Example screenshot of the CMTR website.

My Trauma Recovery (MTR) website is a self-help trauma intervention program based on social cognitive theory [17], which consists of six modules of social support, self-talk, relaxation, trauma triggers, unhelpful coping, and professional help [7,18]. It has been translated, as CMTR, by funding via a Swiss-Chinese collaboration between University of Zurich (A Maercker) and Beijing Normal University (J Wang). The translation work was done mainly by the first author, and the second author (and her master’s students) and the third author (and his doctoral students) were involved in the back-translation work. CMTR utilizes interactive components, such as pictures, audio segments, video segments, and self-tests, to offer educational information on trauma and provide trauma coping skills practice for its users. All pictures on the CMTR website were new ones with Chinese figures; in addition, a total of 27 audio segments on the website were newly created. Due to the high costs of video, five video segments were kept in English with Chinese subtitles added to these videos. The users are encouraged to take self-tests regularly on CMTR so that they will receive a series of updated charts on their post-traumatic distress, depression symptoms, social support perceptions, and coping self-efficacy levels. An example screenshot of the CMTR website is given in Figure 1.

### Participants and Procedure

#### Overview

Participants were recruited through two main channels from November 2011 to August 2012 and they completed follow-up tests before the end of January 2013. This study was approved by the research ethics board at Beijing Normal University. The urban sample was reached through Internet advertisements and participants were contacted only by email during the research period. The rural sample was recruited in-person via cooperation with a counseling center in Beichuan county in Sichuan province, where a severe earthquake occurred in May 2008; they were supported by volunteers with Internet access and minimally reimbursed for their participation. We expected small effect sizes of 0.2 in the main analyses, with a sample size of 139 [19] (the sample size was limited by funding restrictions). In each sample, the participants were randomly assigned to the treatment or waiting list condition based on a computer-generated randomization list. Assessment had been computer-generated on a professional Chinese survey website (equals a blinded assessment).

The criteria for inclusion were as follows: (1) experienced at least one traumatic event according to the Diagnostic and Statistical Manual of Mental Disorders, fourth edition (DSM-IV) [20] trauma criteria, (2) the latest traumatic event happened 3-60 months prior, and (3) the person reported at least two PTSD symptoms in the trauma screening questionnaire. Respondents were excluded if they had insufficient reading or auditive comprehension competency in the Chinese language, insufficient Internet access time (<360 minutes in 4 weeks), acute psychotic symptoms, or were receiving other mental health intervention.

#### Urban/Unsupported Sample

**Figure 2:**
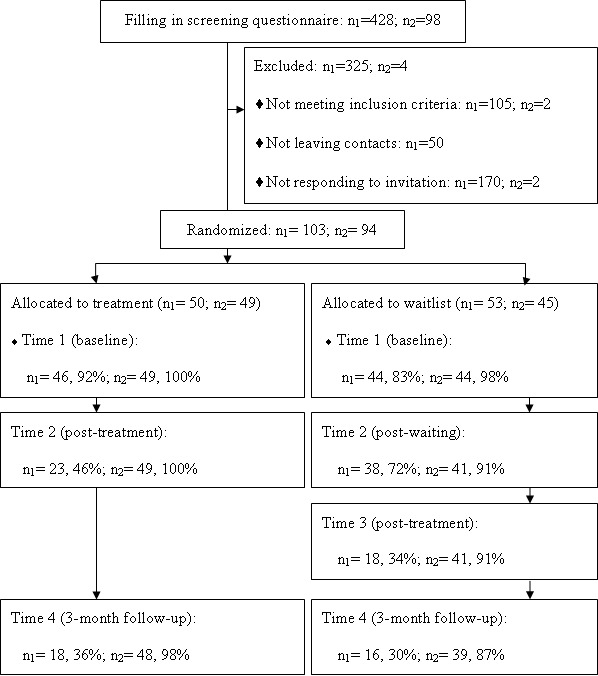
Participant flow in this study. n_1_=number of participants in the urban sample; n_2_=number of participants in the rural sample.

Research assistants put advertisements for the research program online via online bulletins, blogs, microblogs, and personal websites. They also distributed flyers with CMTR website information through private contacts at 10 university/hospital counseling centers. The advertisements recruited persons who had experienced traumatic events within the last five years, had suffered from tense distress since then, and had an interest in reducing their distress through a self-help intervention program. As shown in Figure 2, a total of 428 people responded to the advertisements, among which at least 80% were reached via online bulletins, blogs, microblogs, and personal websites. All 428 people were invited via the advertisements to fill in trauma and psychosis screening questionnaires online. Research assistants gave feedback on screening results to people who left contact information and sent a research invitation to those who were eligible for the program. When a person read the participant information and returned a signed consent form by email, he or she was accepted as a participant and his or her sequence number was used as the participant ID. According to a random numbers list, the participants were randomly allocated to one of the two groups.

All participants first completed a baseline test (Time 1) online. Those in the treatment group received a user account to start the one-month intervention on the CMTR website, while those in the waiting list group had to wait for one month. One month later, both groups completed the post-treatment/waiting test (Time 2). The participants in the waiting list group then started treatment with their user accounts and filled out the post-treatment test (Time 3) one month later. All of the participants finished the follow-up test (Time 4) three months after the completion of the online treatment. The participants were encouraged to use the CMTR website as often as possible at the beginning of the treatment period and they decided themselves when, where, and how often to use the website during the one-month period.

#### Rural/Supported Sample

To recruit participants, the cooperative counseling center (Zhong Ke Bo Ai, Institute of Psychological Medicine) made research invitation phone calls to known earthquake survivors on their previously collected list. Due to lack of Internet service at home in Beichuan, all participants had to complete tests and receive online treatment in the counseling center’s computer room. At the beginning, volunteers gave information about payment for participation. On average, a participant got a total pay of US$58 in kind (eg, rice, cooking oil, pot, etc), if he or she completed the research procedure. All participants were paid progressively more after each visit to the counseling center.

After a face-to-face screening, eligible participants were randomly assigned to the treatment or waiting list group. During the one-month treatment, participants visited the center every 5 days to use CMTR for at least half an hour (5 times). The post-assessment (Time 2) also took place at the center. The participants on the waiting list started the treatment after a one-month delay. Three months after the completion of online treatment, the two groups filled out a follow-up test (Time 4) at the center.

Assistant volunteers were instructed to provide support only with technical problems on the CMTR website. When participants asked for help with their mental problems or website contents, they received a brief reply that CMTR was a self-help program, they could learn to cope with their problems on the website, and they would get further information on mental health help, if needed, after Time 4.

### Measures

#### Trauma and Psychosis Screening Questionnaires

A list of 12 traumatic event types was adopted from the MTR website. Participants chose one or more events that they had experienced recently and reported the date of the latest traumatic event. The 10-item Trauma Screening Questionnaire (TSQ) [21] was used to measure PTSD symptoms among the first 71 respondents in the urban sample and the 7-item Short Screening Scale for DSM-IV PTSD [22] was then substituted for the TSQ. This substitution was done because the 7-item Short Screening Scale has more comprehensive coverage of symptom groups with fewer items.

Five items for psychotic symptoms were taken from the German Diagnostic Interview for Psychiatric Symptoms (DIPS) [23]. The DIPS covers all affective, anxiety, and somatoform disorders based on Diagnostic and Statistical Manual of Mental Disorders, fourth edition, text revision (DSM-IV-TR) [24] and screens for psychosis. It has excellent reliability and validity values [23].

#### Trauma-Related Distress Questionnaires

##### Primary Outcome Measure / Post-Traumatic Diagnostic Scale (PDS)

This scale includes 17 PTSD symptom items assessing the frequency of trauma-related symptoms in the past month on a 4-point scale (0=not at all or only one time, 3=five or more times a week/almost always) [25]. Its Chinese version has good psychometric properties in Taiwan samples [26]. The internal consistency of the scale in this study was measured at Cronbach alpha=.92.

##### Secondary Outcome Measures / Symptom Checklist 90-Depression (SCL-D)

The 13-item depression subscale of SCL [27] was used to measure to what extent participants had been bothered by depressive symptoms in the past month on a 5-point scale, ranging from 0 (not at all) to 4 (extremely). Its Chinese version has been tested in various Chinese samples and shows good psychometric properties [28]. The internal consistency of the scale in this study was Cronbach alpha=.94.

##### Post-Traumatic Cognitive Changes (PCC)

Five items were adopted from the MTR website to indicate participants’ cognitive changes (feeling guilty, worrying about bad things, feeling permanently harmed, and going crazy) after traumatic experiences. Example items are: “I now believe that the world is a very dangerous place”, and “I have been permanently harmed (not considering any physical injuries sustained) by the event.” A 5-point scale was used ranging from 0 (not at all) to 4 (extremely). The internal consistency of the questionnaire in this study was Cronbach alpha=.84.

##### Social Functioning Impairment (SFI)

Four questions were adopted from the MTR website to examine participants’ functional impairment (ie, not able to complete normal responsibilities, disturbing relationships with family or friends, not able to go out and spend time with friends, not able to do other activities the person would like to be doing) after trauma experiences. Example questions are: “To what extent have your reactions to what has happened reduced your ability to complete your normal responsibilities (eg, job, school, home, childcare duties)?” and “How much have these reactions disturbed your relationships with your family or friends?” Participants answered the questions on a 5-point scale (0=not at all, 4=extremely). The internal consistency of the questionnaire in this study was Cronbach alpha=.88.

##### Trauma Coping Self-Efficacy Scale (CSE)

This 10-item scale is a short version of the CSE for Trauma [18]. It measures to what extent participants felt capable of coping with PTSD reactions at different assessment points. A 5-point scale was used ranging from 0 (not at all) to 4 (extremely). The internal consistency of the scale in this study was Cronbach alpha=.83.

### Data Analyses

General Linear Model (GLM) was used to examine group×time interactions for all outcome measures from Time 1 to Time 2 within each sample. A series of subsequent ANOVAs (analysis of variance) was then applied. First, within each sample between-group comparisons were made for the two conditions (intervention vs waiting list) at the subsequent points in time (Time 1 to 4, as explained in Figure 2). Second, in each sample we applied within-group comparisons for time effects. Due to the high dropout rates in the urban sample, we decided to apply an intend-to-treat analysis (ITT; last value carried forward). For further analyses on dropouts in the urban sample, see Wang et al [29].

## Results

### Demographic Statistics and Traumatic Experiences

Demographic statistics of the 183 participants at Time 1 for each sample are presented in Table 1. Within each sample, the treatment group did not differ from the waiting list group in the five demographic characteristics.

In the urban sample, 51% (46/90) participants reported two or more types of traumatic events. Most frequently reported trauma types were physical assault (29/90, 32%), a sudden and unexpected death of someone close (26/90, 29%), a serious accident at work, home, or during recreational activity (15/90, 17%), and sexual assault (13/90, 14%). Among the 90 participants, 27% (24/90) experienced their latest trauma within 3 months, 60% (54/90) between 3-60 months, and 12% (11/90) longer than 60 months. In the rural sample, 86% (80/93) participants reported two or more types of trauma events. Most common trauma types were natural disasters (92/93, 99%), a sudden and unexpected death of someone close (69/93, 74%), a sudden and violent death of another person (40/93, 43%), and physical assault (40/93, 43%). Concerning the date of the latest trauma, all participants (93/93, 100%) reported 3-60 months prior.

The two samples did not differ on PDS at Time 1 (urban sample: mean 29.43, SD 10.19; rural sample: mean 30.22, SD 8.88; *F*_1,181_=0.31, *P*=.58). Using the cut-offs for symptom severity rating of 0 (no rating), 1-10 (mild), 11-20 (moderate), 21-35 (moderate to severe), and 36-51 (severe) [31], 24% (22/90) of participants in the urban sample reported (1-20) mild to moderate, 44% (40/90) reported (21-35) moderate to severe, and 31% (28/90) reported (36-51) extreme symptom severity. The figures were 9% (8/93), 66% (61/93), and 26% (24/93) for the rural sample, respectively.

As shown in Figure 1, 36% (18/50) of participants in the treatment group and 30% (16/53) of participants in the waiting list group completed all measurements in the urban sample. The figures were 98% (48/49) and 87% (39/45) in the rural sample, respectively. Within each sample, the completers did not differ from the non-completers in any of the five demographic characteristics. One-way ANOVAs showed no significant difference between the completers and the non-completers in PDS, PCC, SFI, SCL-D, and CSE within each sample (for the urban sample: *F*_1,88_=0.08-1.45; all *P*s>.23; for the rural sample: *F*_1,91_=0.18-2.06; all *P*s>.15).

### Treatment Effects

#### Summary

Means and standard deviations for all outcome measures at each assessment time are presented in Table 2. In the urban sample, GLM analyses showed significant group-by-time interactions on PDS (*F*_1,88_=7.65, *P*=.007), PCC (*F*_1,88_=5.32, *P*=.02), and SCL-D (*F*_1,88_=8.37, *P*=.005), but not on SFI (*F*_1,88_=3.33, *P*=.07) and CSE (*F*_1,88_=0.03, *P*=.87). In the rural sample, the group-by-time interaction was significant on PDS (*F*_1,91_=5.35, *P*=.02), but not on the other four outcome measures (*F*_1,91_=0.01-1.12, all *P*s>.29).

#### Between-Group Differences

Based on one-way ANOVAs (Table 2), in each sample, the treatment group did not differ from the waiting list group on all measures at Time 1. After the one-month treatment (Time 2), this group scored significantly lower than the waiting list group on PDS, PCC, SFI, and SCL-D in the urban sample. In the rural sample, the group difference was significant only on PDS at Time 2.

At 3-month follow-up measurement, the ITT analysis revealed no significant between-group difference in either sample. When the two samples were compared, they differed significantly at Time 4 on SFI (*F*_1,181_=6.97, *P*=.009) and CSE (*F*_1,181_=7.19, *P*=.008), but did not differ significantly on PDS, PCC, and SCL-D (*F*_1,181_=0.34-1.05; all *P*s>.30).

#### Within-Group Differences

In the urban sample, as presented in Table 3, the treatment group showed significant improvement on PDS, PCC, SFI, and SCL-D from Time 1 to Time 2, and the improvement was sustained during the follow-up period. Within-group evaluations showed no additional improvement or decrease on five measures from Time 3 to Time 4 (*F*_1,45_=0.02-3.04; all *P*s>.08; *d*=0.03-0.38). During the one-month waiting period, the waiting list group remained stable on five measures. After the completion of delayed treatment, the waiting list group showed nearly significant improvement on PDS (*P*=.053) and significant decrease on PCC from Time 2 to Time 3. The improvement continued during the follow-up period. Further, within-group comparisons revealed significant decrease on PDS (*F*_1,43_=10.63, *P*=.002, *d*=0.70), PCC (*F*_1,43_=11.00, *P*=.002, *d*=0.71), SFI (*F*_1,43_=10.04, *P*=.003, *d*=0.68), and SCL-D (*F*_1,43_=10.33, *P*=.002, *d*=0.69), but not significant increase on CSE (*F*_1,43_=3.27, *P*=.08, *d*=0.39) from Time 3 to Time 4.

In the rural sample, the treatment group also reported significant improvement on PDS, PCC, SFI, and SCL-D from Time 1 to Time 2. The improvement on SCL-D became, however, non-significant three months later. Further, within-group evaluations showed no change on five measures from Time 3 to Time 4 (*F*_1,48_=0.01-1.06; all *P*s>.30; *d*=0.01-0.21). For the waiting list group, no change occurred on PDS, PCC, and CSE, but significant decrease appeared on SFI and SCL-D from Time 1 to Time 2. After one-month delayed treatment, the group scored significantly lower on PDS and SCL-D at Time 3 than Time 2. The improvement disappeared, however, at Time 4. Based on further within-group comparison results, the group showed significant increase on SCL-D (*F*_1,43_=5.20; *P*=.03; *d*=0.49) and non-significant change on the other four measures (*F*_1,43_=0.24-1.72; all *P*s>.19; *d*=0.10-0.28) from Time 3 to Time 4.

**Table 1:** Demographic characteristics for the urban sample (n=90) and the rural sample (n=93).

		Urban sample n (%)	Rural sample n (%)
**Gender**			
	Female	67 (74)	76 (82)
	Male	23 (26)	17 (18)
**Age**			
	18-	6 (7)	
	18-25	40 (44)	1 (1)
	26-40	36 (40)	42 (45)
	41-55	8 (9)	37 (40)
	56-70		13 (14)
**Family income per year ($)** ^a^			
	0-10,000	58 (64)	89 (96)
	10,001-20,000	19 (21)	1 (1)
	20,001+	7 (8)	1 (1)
**Marital status**			
	Single	64 (71)	5 (5)
	Married	24 (27)	88 (95)
**Education**			
	Junior middle school/lower	1 (1)	67 (72)
	High middle school	10 (11)	19 (20)
	Bachelor’s degree	62 (69)	7 (8)
	Master’s degree/higher	17 (19)	

^a^According to statistics published on the National Bureau of Statistics of China website on January 18, 2013, the annual per capita net income was about US$1,319 for rural households and about US$4,094 for urban households in 2012 [30].

**Table 2:** Descriptive statistics, between-group comparisons, and effect sizes (Cohen’s *d*) for each sample: intention-to-treat analysis.

			Treatment group mean (SD)		Waiting list group mean (SD)	*F*_1,88_ (Cohen’s *d*)		*P*
**Urban sample**								
	**Time 1**							
		PDS^a^		1.74 (0.61)	1.73 (0.59)	0.01 (0.02)	.95	
		PCC^b^		2.54 (0.87)	2.78 (0.85)	1.78 (−0.28)	.19	
		SFI^c^		2.66 (1.09)	2.79 (0.92)	0.38 (−0.13)	.54	
		SCL-D^d^		2.64 (0.79)	2.66 (0.87)	0.01 (−0.02)	.92	
		CSE^e^		1.99 (0.73)	1.88 (0.56)	0.67 (0.17)	.42	
	**Time 2**							
		PDS		1.13 (0.73)	1.65 (0.58)	4.29 (−0.44)	.04	
		PCC		1.77 (1.09)	2.69 (0.86)	8.50 (−0.61)	.005	
		SFI		1.84 (0.96)	2.62 (0.78)	5.08 (−0.48)	.03	
		SCL-D		1.71 (0.86)	2.52 (0.91)	4.32 (−0.44)	.04	
		CSE		2.25 (0.74)	2.04 (0.72)	0.79 (0.19)	.38	
	**Time 3**							
		PDS			1.33 (0.75)			
		PCC			2.29 (0.99)			
		SFI			2.33(1.19)			
		SCL-D			2.14 (1.03)			
		CSE			2.21 (0.70)			
	**Time 4**							
		PDS		0.76 (0.78)	0.74 (0.58)	0.18 (0.09)	.67	
		PCC		1.39 (1.11)	1.43 (0.87)	0.44 (−0.14)	.51	
		SFI		1.60 (1.27)	1.58 (1.08)	0.89 (0.20)	.35	
		SCL-D		1.24 (1.01)	1.39 (0.69)	0.27 (−0.11)	.60	
		CSE		2.34 (1.15)	2.74 (0.49)	0.41 (−0.14)	.52	
**Rural sample**								
	**Time 1**							
		PDS		1.77 (0.48)	1.79 (0.57)	0.05 (−0.04)	.83	
		PCC		2.53 (0.84)	2.35 (0.88)	1.07 (0.21)	.30	
		SFI		2.31 (0.95)	2.49 (0.90)	0.96 (−0.19)	.33	
		SCL-D		2.17 (0.77)	2.32 (0.95)	0.63 (−0.17)	.43	
		CSE		1.96 (0.54)	1.97 (0.63)	0.01 (−0.02)	.97	
	**Time 2**							
		PDS		1.34 (0.48)	1.62 (0.55)	6.86 (−0.54)	.01	
		PCC		2.09 (0.84)	2.11 (0.85)	0.001 (−0.01)	.97	
		SFI		1.85 (0.77)	1.88 (0.94)	0.09 (−0.06)	.77	
		SCL-D		1.92 (0.66)	2.08 (0.86)	0.96 (−0.20)	.33	
		CSE		1.96 (0.39)	1.84 (0.52)	1.60 (0.26)	.21	
	**Time 3**							
		PDS			1.38 (0.56)			
		PCC			1.86 (0.82)			
		SFI			1.70 (0.86)			
		SCL-D			1.68 (0.80)			
		CSE			1.68 (0.58)			
	**Time 4**							
		PDS		1.37 (0.58)	1.54 (0.63)	1.85 (−0.28)	.18	
		PCC		2.02 (0.94)	1.96 (1.02)	0.15 (0.08)	.70	
		SFI		1.73 (0.90)	1.83 (0.88)	0.21 (−0.10)	.65	
		SCL-D		1.97 (0.93)	2.08 (0.96)	0.11 (−0.07)	.74	
		CSE		1.96 (0.53)	1.86 (0.72)	1.28 (0.23)	.26	

^a^PDS: Post-traumatic Diagnostic Scale

^b^PCC: Post-traumatic Cognitive Changes questionnaire

^c^SFI: Social Functioning Impairment questionnaire

^d^SCL-D: Symptom Checklist 90-Depression scale

^e^CSE: Trauma Coping Self-Efficacy scale

**Table 3:** Within-groups comparisons and effect sizes (Cohen’s *d*) for each sample: intention-to-treat analysis.

			Treatment group		Waiting list group	
			*F*_1,45_ (Cohen’s *d*)	*P*	*F*_1,43_ (Cohen’s *d*)	*P*
**Urban sample**						
	**Time 1 vs 2**					
		PDS^a^	15.13 (0.81)	<.001	0.77 (0.19)	.39
		PCC^b^	13.86 (0.78)	.001	1.46 (0.26)	.23
		SFI^c^	11.08 (0.69)	.002	2.71 (0.35)	.11
		SCL-D^d^	19.69 (0.93)	<.001	1.91 (0.29)	.17
		CSE^e^	3.36 (−0.38)	.07	1.76 (−0.28)	.19
	**Time 2 vs 3**					
		PDS			3.97 (0.42)	.053
		PCC			6.45 (0.54)	.02
		SFI			0.62 (0.17)	.44
		SCL-D			2.44 (0.33)	.13
		CSE			1.46 (−0.26)	.23
	**Time 1/2** ^f^ ** vs 4**					
		PDS	17.29 (0.87)	<.001	14.57 (0.81)	<.001
		PCC	13.31 (0.76)	.001	12.69 (0.76)	.001
		SFI	13.80 (0.77)	.001	7.18 (0.57)	.01
		SCL-D	20.61 (0.95)	<.001	14.79 (0.82)	<.001
		CSE	1.34 (−0.24)	.25	6.42 (−0.54)	.02
**Rural sample**						
	**Time 1 vs 2**					
		PDS	43.97 (1.34)	<.001	3.68 (0.41)	.06
		PCC	13.64 (0.75)	.001	3.38 (0.39)	.07
		SFI	15.42 (0.79)	<.001	13.95 (0.80)	.001
		SCL-D	5.78 (0.49)	.02	5.12 (0.48)	.03
		CSE	0.001(0.01)	.98	1.71 (0.28)	.20
	**Time 2 vs 3**					
		PDS			4.42 (0.45)	.04
		PCC			2.83 (0.36)	.10
		SFI			0.95 (0.21)	.34
		SCL-D			7.27 (0.58)	.01
		CSE			2.56 (0.34)	.12
	**Time 1/2** ^f^ ** vs 4**					
		PDS	24.22 (0.99)	<.001	1.10 (0.22)	.30
		PCC	16.41 (0.82)	<.001	1.82 (0.29)	.19
		SFI	16.85 (0.83)	<.001	0.41 (0.14)	.52
		SCL-D	2.99 (0.35)	.09	0.17 (0.09)	.68
		CSE	0.002 (−0.01)	.97	0.04 (0.04)	.85

^a^PDS: Post-traumatic Diagnostic Scale

^b^PCC: Post-traumatic Cognitive Changes questionnaire

^c^SFI: Social Functioning Impairment questionnaire

^d^SCL-D: Symptom Checklist 90-Depression scale

^e^CSE: Trauma Coping Self-Efficacy scale

^f^Time 1 vs 4 for treatment groups, Time 2 vs 4 for waiting list groups

## Discussion

### Principal Findings

This study aims to examine the efficacy of a Chinese self-help intervention program (CMTR) for traumatized persons. Its English version (MTR) had been empirically examined in a US sample of 56 Hurricane Ike survivors and showed effectiveness in reducing participants’ worry and depression level [18]. This study tested CMTR to parallel RCTs in one urban/unsupported sample and one rural/supported sample. The former sample consisted of an urban sample. Most of them were younger than 40 years old, single, with a bachelor’s or higher degree, and a low to middle family income level [30]. It covers main characteristics of Internet users in China [32]. A parallel sample came from a rural area using the advantage of Web-based interventions for offering mental health services for people far away from urban areas. It also tried to address problems of Internet supply in populations with many elderly and lower-educated people by providing them with IT access and support.

The CMTR program showed significant effectiveness in reducing participants’ PTSD symptom severity during the one-month treatment/waiting period in the two samples. The program also produced significant improvement of other mental health outcomes (post-traumatic cognitive changes, functional impairment, and depression) after controlling time effects in the urban/unsupported sample by the applied design. These findings give support for the short-term efficacy of CMTR [18] in the two Chinese populations and contribute to the literature that self-help Web-based programs can be used to provide mental health help for traumatized persons [6,33]. Different from the sample with minimal presence of PTSD symptoms in Steinmetz et al [18], more than two-thirds of the participants in this study reported moderate or severe PTSD symptom severity.

After the two waiting list groups completed one-month delayed treatment, they showed improvement with moderate effect sizes on PTSD symptoms and post-traumatic cognitive changes/depression level—converging with the favorable effect sizes of the main trial. Regarding the group comparison, two findings call for attention. The first is that the waiting list group showed a lower dropout rate than the treatment group at post-treatment/waiting test in the urban sample. Participants’ motivation to use the CMTR website may be one potential factor to understand this finding. In the urban sample, all participants took part actively in the program through Internet advertisements and completed the research procedure without any payment. Thus, the participants may have been highly motivated to follow the research instructions before they were able to use the CMTR website, and their high level of motivation may have decreased after using the CMTR website. Based on our data, after the one-month delayed treatment, 18 out of 38 participants (47%) in the waiting list group who used the website completed post-test, which is very similar to the proportion of participants who completed post-test in the treatment group (23/46, 50%).

Previous studies have shown that self-help intervention programs are most efficient for motivated users in the treatment of anxiety disorders [34]. In the current study, we found bigger pre-post intervention differences in the treatment group than in the waiting list group in the urban sample, although the latter may have higher levels of motivation prior to using the treatment program. However, the waiting list group showed significant additional improvement on four outcome measures while the treatment group remained stable on all outcome measures during the three-month follow-up period. Further research is needed to examine the influence of motivation on Web-based intervention efficacy, particularly over the long term.

The second finding is, without any treatment, the waiting list group reported significant decrease on social functioning impairment and depression symptoms after one-month waiting period in the rural sample. They also reported lower level of PTSD symptoms and post-traumatic cognitive changes with moderate effect sizes. Such a placebo effect may be explained by the participants’ face-to-face contact with well-known professionals. Because most participants in the rural sample were quite unfamiliar with Internet service, the volunteers at the center helped them to log in to CMTR to use the website. The participants could ask for help from volunteers about Internet service problems at the website, but they did not receive help with the contents on CMTR. For example, these volunteers did not give advice on which content on CMTR to learn first, how much content to finish during one treatment session, or read/explain certain content for the participants. However, such face-to-face contact still influenced the treatment effect of the CMTR program in addition to its impact on the dropout rates in the rural sample.

In the current study, the self-help CMTR website was thus less effective in the rural sample than in the urban sample. Controlling the time (placebo) effect, the treatment group showed significant improvement only on PTSD symptom severity than the waiting list group in the rural sample. After one-month delayed treatment, the waiting list group from rural areas showed further decrease on PTSD and depression symptoms, but the pre-post intervention differences disappeared during three-month follow-up period. The efficacy difference between the two samples may be due to the participants’ lower level of motivation in the rural sample. These participants participated in the program in a more passive way (having been recruited and subsequently supported with their Web use by the center) and they received payment for completion of every test. Thus, they may have followed the research instructions because of the reward and were less motivated to use the CMTR website than the participants in the urban sample. Also, the rural sample in this study may have benefited less from the CMTR website due to their lack of Internet access or knowledge. Based on the feedback from the volunteers, many participants, particularly the elderly, read slowly through the website. It is thus optimistic to expect better treatment efficacy of the CMTR website in non-Internet user populations, when the users would get (minimal) guidance.

In addition, neither sample in this study showed significant improvement in coping self-efficacy. Steinmetz et al [18] argued that the moderate presence of CSE level in their sample may cause the MTR website aspects targeted at increasing CSE to be less relevant to participants’ needs. In the current samples, participants also reported moderate to high CSE mean scores at the baseline test. Further studies need to test the efficacy of the CMTR program in enhancing users’ coping ability and to explain its effectiveness in reducing users’ PTSD symptom severity in cross-cultural comparison.

### Limitations

The current study has limitations in sampling and in controlling the contact between research assistants/volunteers and participants. Future studies need to examine the CMTR website in a larger, representative sample. Given that the current study used self-selected samples, the findings cannot be generalized to populations from hospitals or outpatient clinics. Also, it is important to detect if the efficacy of the CMTR website will remain long term in the treatment of PTSD.

### Conclusions

The current study provides preliminary support for the short-term treatment efficacy of the CMTR website in two modalities of application. For those traumatized people who have good access to Internet service, the website may be an effective self-help intervention program for their trauma recovery. For those who are in need of treatment but lack Internet knowledge, the CMTR website may be also an effective intervention tool that can be used easily with minimal guidance. However, further research is needed to examine the program’s long-term efficacy in large samples and explore the influence of different application modalities (eg, involvement of mental health professionals) on the program’s usage (eg, dropout rate, treatment effect).

## Multimedia Appendix 1

CONSORT-EHEALTH checklist V1.6.2 [35].

## Abbreviations

CMTR: Chinese version of My Trauma Recovery program
CSE: Trauma Coping Self-Efficacy Scale
DIPS: German Diagnostic Interview for Psychiatric Symptoms
DSM-IV: Diagnostic and Statistical Manual of Mental Disorders, fourth edition
GLM: General Linear Model
MTR: My Trauma Recovery program
PCC: Post-traumatic Cognitive Changes
PDS: Post-traumatic Diagnostic Scale
PTSD: post-traumatic stress disorder
SCL-D: Symptom Checklist 90-Depression
SFI: Social Functioning Impairment
TSQ: Trauma Screening Questionnaire
