# Supplementary material for: Chinese My Trauma Recovery, A Web-Based Intervention for Traumatized Persons in Two Parallel Samples: Randomized Controlled Trial
Source: J Med Internet Res. 2013 Sep 30;15(9):e213. doi: 10.2196/jmir.2690 (PMC3815433; doi:10.2196/jmir.2690)
Supplement: Supplementary file 1 [file jmir_v15i9e213_app1.pdf]

CONSORT-EHEALTH Checklist V1.6.2 Report

(based on CONSORT-EHEALTH V1.6), available at [http://tinyurl.com/consort-ehealth-v1-6].

2690

Date completed

4/26/2013 11:43:56

by

Zhiyun

Chinese My Trauma Recovery: A combined Web-based Randomized Controlled Trial for Traumatized Persons in Two Parallel Samples

TITLE

1a-i) Identify the mode of delivery in the title

Yes. The title contains "Web-based".

1a-ii) Non-web-based components or important co-interventions in title

1a-iii) Primary condition or target group in the title

Yes. The title contains "Traumatized Persons".

ABSTRACT

1b-i) Key features/functionalities/components of the intervention and comparator in the METHODS section of the ABSTRACT

Yes. The abstract contains "guided self-help interventions for PTSD", "website intervention", and "self-help Web-based programs".

1b-ii) Level of human involvement in the METHODS section of the ABSTRACT

Yes. The abstract contains "self-help Web-based programs".

1b-iii) Open vs. closed, web-based (self-assessment) vs. face-to-face assessments in the METHODS section of the ABSTRACT

Yes. The abstract contains "Assessment was completed online at a professional Chinese survey website."

1b-iv) RESULTS section in abstract must contain use data

No. Use data were reported in another submitted paper. In this paper, use data were presented in participant flow diagram (Figure 2).

1b-v) CONCLUSIONS/DISCUSSION in abstract for negative trials

No. Primary outcome changed in this paper.

INTRODUCTION

2a-i) Problem and the type of system/solution

Yes. "The current study aims to build a Chinese Web-based self-guided intervention program for traumatized persons and to test its effectiveness in Chinese populations."

2a-ii) Scientific background, rationale: What is known about the (type of) system

Yes.

"In recent years the Internet has been adopted as a valuable tool to deliver mental health services for large populations [3-4]. Different Internet-based intervention programs have been developed to help people recover from PTSD [5]....These programs have been examined in American and European countries and have shown significant effects in reducing people's traumatic stress-related distress [8, 10]. However, few programs have been developed for and tested in Asian populations. "

"[In China] few people got help from mental health professionals to deal with their trauma related problems [13]. A major obstacle to people's mental health help seeking behavior is the unavailability of professionals in China, especially in rural areas [14]. The number of qualified mental health professionals is small, even in large cities like Beijing and Shanghai [15]. ...The Internet thus offers a useful way to improve mental health services for people after trauma in China."

METHODS

3a) CONSORT: Description of trial design (such as parallel, factorial) including allocation ratio

Yes.

"To test the effectiveness of the Chinese My Trauma Recovery (CMTR) program, the current study adopted a randomized controlled pre-, post-, and 3-month follow-up trial design in a two-arm design (urban/unsupported vs. rural/supported). It was expected that participants from the two treatment groups would show significant improvement in PTSD symptoms and general mental health compared to the respective waiting list groups. Explorative post-hoc analyses compared the effect sizes of the two arms of the study (urban/unsupported vs. rural/supported)."

3b) CONSORT: Important changes to methods after trial commencement (such as eligibility criteria), with reasons

No changes to methods after trial commencement.

3b-i) Bug fixes, Downtimes, Content Changes

4a) CONSORT: Eligibility criteria for participants

Yes.

"The criteria for inclusion were: 1) experienced at least one traumatic event according to Diagnostic and Statistical Manual of Mental Disorders, fourth edition (DSM-IV) [20] trauma criteria; 2) the latest traumatic event happened 3 to 60 months before; 3) the person reported at least two PTSD symptoms in the trauma screening questionnaire."

4a-i) Computer / Internet literacy

This paper used data from a rural sample, consisting of participants with low education and little computer knowledge, in addition to an urban sample. Thus, respondents were excluded when "he/she had insufficient reading or auditive comprehension competency of Chinese language".

4a-ii) Open vs. closed, web-based vs. face-to-face assessments:

Yes.

"The urban sample was reached through Internet advertisements, and participants were contacted only by email during the research period. The rural sample was recruited face to face via cooperation with a counseling center in Beichuan county in Sichuan province, where a severe earthquake occurred in May 2008; they were supported by volunteers with the Internet access and minimally reimbursed for their participation... Assessment had been computer-generated at a professional Chinese survey website (equals a blinded assessment)."

4a-iii) Information giving during recruitment

4b) CONSORT: Settings and locations where the data were collected

Yes.
"Participants were recruited through two main channels from November 2011 to August 2012 and they completed follow-up tests before the end of January 2013. The urban sample was reached through Internet advertisements, and participants were contacted only by email during the research period. The rural sample was recruited face to face via cooperation with a counseling center in Beichuan county in Sichuan province, where a severe earthquake occurred in May 2008."

4b-i) Report if outcomes were (self-)assessed through online questionnaires

Yes.
"Assessment had been computer-generated at a professional Chinese survey website (equals a blinded assessment)."

4b-ii) Report how institutional affiliations are displayed

5) CONSORT: Describe the interventions for each group with sufficient details to allow replication, including how and when they were actually administered

5-i) Mention names, credential, affiliations of the developers, sponsors, and owners

Yes.
"MTR website is a self-help trauma intervention program based on social cognitive theory [17], which consists of six modules of social support, self-talk, relaxation, trauma triggers, unhelpful coping and professional help [7, 18]. It has been translated as CMTR by means of funding of a Swiss-Chinese Collaboration project between University of Zurich (A. Maercker) and Beijing Normal University (J. Wang)."

5-ii) Describe the history/development process

5-iii) Revisions and updating

5-iv) Quality assurance methods

5-v) Ensure replicability by publishing the source code, and/or providing screenshots/screen-capture video, and/or providing flowcharts of the algorithms used

Yes. See Figure 1.

5-vi) Digital preservation

5-vii) Access

Yes.
"All participants first completed a baseline test (Time 1) online. Those in the treatment group received a user account to start the one-month intervention at the CMTR website, while those in the waiting list group had to wait for one month. One month later, both groups completed the post-treatment/waiting test (Time 2). The participants in the waiting list group then started treatment with their user accounts and filled out the post-treatment test (Time 3) one month later. All of the participants finished the follow-up test (Time 4) three months after the completion of the online treatment. The participants were encouraged to use the CMTR website as often as possible at the beginning of the treatment period, and they decided themselves when, where and how often to use the website during the one month period."

5-viii) Mode of delivery, features/functionalities/components of the intervention and comparator, and the theoretical framework

Yes.
"MTR website is a self-help trauma intervention program based on social cognitive theory [17], which consists of six modules of social support, self-talk, relaxation, trauma triggers, unhelpful coping and professional help [7, 18]. It has been translated as CMTR by means of funding of a Swiss-Chinese Collaboration project between University of Zurich (A. Maercker) and Beijing Normal University (J. Wang). CMTR utilizes interactive components, such as pictures, audio segments, video segments and self-tests, to offer educational information on trauma and provide trauma coping skills practice for its users. All pictures on the CMTR website were new ones with Chinese figures; in addition, a total of twenty-seven audio segments at the website were newly created. Due to high costs of videos, five video segments were kept in English with Chinese subtitles added to these videos. The users are encouraged to take self-tests regularly at CMTR so that they will receive a series of updated charts on their posttraumatic distress, depression symptoms, social support perceptions, and coping self-efficacy levels."

5-ix) Describe use parameters

Yes.
In the urban sample: "The participants were encouraged to use the CMTR website as often as possible at the beginning of the treatment period, and they decided themselves when, where and how often to use the website during the one month period."
In the rural sample: "During the one-month treatment participants visited the center every five days to use CMTR for at least half an hour (5 times)."

5-x) Clarify the level of human involvement

Yes.
"Assistant volunteers were instructed to provide support only with technical problems at the CMTR website. When participants asked for help with their mental problems or website contents, they received a brief reply that CMTR was a self-help program, they could learn to cope with their problems at the website, and they would get further information on mental health help, if needed, after Time 4. "

5-xi) Report any prompts/reminders used

Yes.
"The urban sample was reached through Internet advertisements, and participants were contacted only by email during the research period." The participants received emails informing them to take tests, giving them user accounts information to start treatment and to stop treatment, but they received no reminders of visiting the program during one-month treatment.
In the rural sample: "To recruit participants, the cooperative counseling center (Zhong Ke Bo Ai, Institute of Psychological Medicine) made research invitation phone calls to known earthquake survivors on their previously collected list." The participants were contacted via telephone to take tests and to visit the counseling centre for treatment.

5-xii) Describe any co-interventions (incl. training/support)

No. This paper reported a self-help intervention program application.

6a) CONSORT: Completely defined pre-specified primary and secondary outcome measures, including how and when they were assessed

Yes, like "Trauma-related Distress Questionnaires at Time 1 to 4".

6a-i) Online questionnaires: describe if they were validated for online use and apply CHERRIES items to describe how the questionnaires were designed/deployed

6a-ii) Describe whether and how “use” (including intensity of use/dosage) was defined/measured/monitored

6a-iii) Describe whether, how, and when qualitative feedback from participants was obtained

6b) CONSORT: Any changes to trial outcomes after the trial commenced, with reasons
No changes to trial outcomes after the trial commenced.
7a) CONSORT: How sample size was determined
7a-i) Describe whether and how expected attrition was taken into account when calculating the sample size
Yes.
"We expected small effect sizes of 0.2 in the main analyses, which refers to a sample size of 139 [19]."
7b) CONSORT: When applicable, explanation of any interim analyses and stopping guidelines
Yes.
"When applicable, funding restrictions determined the stopping of sampling."
8a) CONSORT: Method used to generate the random allocation sequence
Yes.
"In each sample, the participants were randomly assigned to the treatment or waiting list condition based on a computer-generated randomization list."
8b) CONSORT: Type of randomisation; details of any restriction (such as blocking and block size)
"In each sample, the participants were randomly assigned to the treatment or waiting list condition based on a computer-generated randomization list."
9) CONSORT: Mechanism used to implement the random allocation sequence (such as sequentially numbered containers), describing any steps taken to conceal the sequence until interventions were assigned
"In each sample, the participants were randomly assigned to the treatment or waiting list condition based on a computer-generated randomization list."
"When a person read the participant information and returned a signed statement of agreement by email, he/she was accepted as a participant and his/her sequence number was used as the participant ID. According to a random numbers list the participants were randomly allocated to the two groups. "
10) CONSORT: Who generated the random allocation sequence, who enrolled participants, and who assigned participants to interventions
"In each sample, the participants were randomly assigned to the treatment or waiting list condition based on a computer-generated randomization list."
11a) CONSORT: Blinding - If done, who was blinded after assignment to interventions (for example, participants, care providers, those assessing outcomes) and how
11a-i) Specify who was blinded, and who wasn't
"Assessment had been computer-generated at a professional Chinese survey website (equals a blinded assessment)."
11a-ii) Discuss e.g., whether participants knew which intervention was the "intervention of interest" and which one was the "comparator"

11b) CONSORT: If relevant, description of the similarity of interventions
Not applicable.
12a) CONSORT: Statistical methods used to compare groups for primary and secondary outcomes
"General Linear Model (GLM) was used to examine group×time interactions for all outcome measures from Time 1 to Time 2 within each sample. A series of subsequent ANOVAs was then applied. First, within each sample between-group comparisons were made for the two conditions (intervention vs. wait-list) at the subsequent points in time (Time 1 to 4, as explained in Figure 2). Second, in each sample we applied within-group comparisons for time effects."
12a-i) Imputation techniques to deal with attrition / missing values
"Due to the usual high drop-out rates for self-help websites as well as the almost non-existing drop-out rate for the supported condition we decided to only apply a per-protocol and not an intend-to-treat analysis (for further analyses on drop-outs in the urban sample see Wang et al., submitted)."
12b) CONSORT: Methods for additional analyses, such as subgroup analyses and adjusted analyses
"A series of subsequent ANOVAs was then applied. First, within each sample between-group comparisons were made for the two conditions (intervention vs. wait-list) at the subsequent points in time (Time 1 to 4, as explained in Figure 2). Second, in each sample we applied within-group comparisons for time effects."

RESULTS

13a) CONSORT: For each group, the numbers of participants who were randomly assigned, received intended treatment, and were analysed for the primary outcome
Use data were presented in the participant flow diagram (Figure 2).
13b) CONSORT: For each group, losses and exclusions after randomisation, together with reasons
It was reported in the participant flow diagram (Figure 2).
13b-i) Attrition diagram
It was reported in the participant flow diagram (Figure 2).
14a) CONSORT: Dates defining the periods of recruitment and follow-up
"Participants were recruited through two main channels from November 2011 to August 2012 and they completed follow-up tests before the end of January 2013. "
14a-i) Indicate if critical "secular events" fell into the study period

14b) CONSORT: Why the trial ended or was stopped (early)
"We expected small effect sizes of 0.2 in the main analyses, which refers to a sample size of 139 [19]. When applicable, funding restrictions determined the stopping of sampling."
15) CONSORT: A table showing baseline demographic and clinical characteristics for each group
It was presented in Table 2.
15-i) Report demographics associated with digital divide issues
It was presented in Table 1.
16a) CONSORT: For each group, number of participants (denominator) included in each analysis and whether the analysis was by original assigned groups
16-i) Report multiple "denominators" and provide definitions
It was reported in another submitted paper.
16-ii) Primary analysis should be intent-to-treat

17a) CONSORT: For each primary and secondary outcome, results for each group, and the estimated effect size and its precision (such as 95% confidence interval)
It was reported in Table 2 and 3.
17a-i) Presentation of process outcomes such as metrics of use and intensity of use

17b) CONSORT: For binary outcomes, presentation of both absolute and relative effect sizes is recommended
Not applicable.
18) CONSORT: Results of any other analyses performed, including subgroup analyses and adjusted analyses, distinguishing pre-specified from exploratory
Not conducted.
18-i) Subgroup analysis of comparing only users

19) CONSORT: All important harms or unintended effects in each group
None.
19-i) Include privacy breaches, technical problems

19-ii) Include qualitative feedback from participants or observations from staff/researchers

DISCUSSION

20) CONSORT: Trial limitations, addressing sources of potential bias, imprecision, multiplicity of analyses
20-i) Typical limitations in ehealth trials
"The current study has limitations in sampling and in controlling the contact between research assistants/volunteers and participants. Future studies need to examine the CMTR website in a larger, representative sample. Given that the current study used self-selected samples, the findings cannot be generalized to populations from hospitals or outpatient clinics. Also, it is important to detect if the efficacy of the CMTR website will remain long-term in the treatment of PTSD."
21) CONSORT: Generalisability (external validity, applicability) of the trial findings
21-i) Generalizability to other populations

21-ii) Discuss if there were elements in the RCT that would be different in a routine application setting

22) CONSORT: Interpretation consistent with results, balancing benefits and harms, and considering other relevant evidence
22-i) Restate study questions and summarize the answers suggested by the data, starting with primary outcomes and process outcomes (use)
"This study aims to examine the efficacy of a Chinese self-help intervention program (CMTR) for traumatized persons. Its English version (MTR) had been empirically examined in a U.S. sample of 56 Hurricane Ike survivors and showed effectiveness in reducing participants' worry and depression level [18]. This study tested CMTR to parallel RCTs in one urban/unsupported sample and one rural/supported sample. ...The CMTR program showed significant effectiveness in reducing participants' PTSD symptom severity in the two samples. The program also produced significant improvement of other mental health outcomes (posttraumatic cognitive changes, functional impairment and depression) after controlling time effects in the urban/unsupported sample by the applied design. These findings give support for the efficacy of CMTR [18] and contribute to the literature that PTSD can be treated effectively via self-help Web-based programs [6, 32]."
22-ii) Highlight unanswered new questions, suggest future research
"Neither sample in this study showed significant improvement in coping self-efficacy. Steinmetz et al. [18] argued that the moderate presence of CSE level in their sample may cause the MTR website's aspects targeted at increasing CSE to be less relevant to participants' needs. In the current samples, participants also reported moderate to high CSE mean scores at the baseline test. Further studies need to test the efficacy of the CMTR program in enhancing users' coping ability and to explain its effectiveness in reducing users' PTSD symptom severity in cross-cultural comparison."

Other information

23) CONSORT: Registration number and name of trial registry
"Trial Registration: ACTRN12611000951954, https://www.anzctr.org.au/Trial/Registration/TrialReview.aspx?id=343399 (Archived by WebCite®at http://www.webcitation.org/6G7WyNODk)"
24) CONSORT: Where the full trial protocol can be accessed, if available
No.
People can search for the website by google, and can access to it for use after they pay some fees.
25) CONSORT: Sources of funding and other support (such as supply of drugs), role of funders
"Acknowledgement
This study was financed by a grant from Sino-Swiss Science and Technology cooperation (IZLCZ3 123910) to Profs A. Maercker and J. Wang. We also acknowledge the excellent cooperation with Dr. C. Benight (University of Colorado at Colorado Springs and Bluesun, Inc) and IT support by Daniel Widmer, ETH Zurich."
X26-i) Comment on ethics committee approval
"It was approved by the school's research ethics board, Beijing Normal University."
x26-ii) Outline informed consent procedures

X26-iii) Safety and security procedures

X27-i) State the relation of the study team towards the system being evaluated
"Declaration of Interest
Dr. Zhiyun Wang coordinated the study during her affiliation with Department of Psychology, Zurich University. The authors have no conflicts of interest."
